# Agronomic and anatomic performance of some soybean genotypes under optimal and water-deficit conditions

**DOI:** 10.3389/fpls.2025.1575180

**Published:** 2025-05-01

**Authors:** Alaa A. Soliman, Manar I. Mousa, Mohamed A. Ibrahim, Khaled A. Baiumy, Shimaa A. Shaaban, Mahmoud M. A. Shabana, Eman N. M. Mohamed, Medhat Rehan, Haitian Yu, Yuhua He

**Affiliations:** ^1^ Food Legumes Research Department, Field Crops Research Institute, Agricultural Research Center, Giza, Egypt; ^2^ Institute of Food Crops, Yunnan Academy of Agricultural Sciences, Kunming, China; ^3^ Agronomy Department, Faculty of Agriculture, Benha Univ., Benha, Egypt; ^4^ Department of Agricultural Botany, Faculty of Agriculture, Cairo University, Giza, Egypt; ^5^ Soils, Water and Environment Research Institute (SWERI), Agricultural Research Center, Giza, Egypt; ^6^ Seed Technology Research Department, Field Crops Research Institute, Agricultural Research Center, Giza, Egypt; ^7^ Department of Plant Production, College of Agriculture and Food, Qassim University, Buraydah, Saudi Arabia

**Keywords:** sustainability, drought, climate change, soybean crop, stem and leaf anatomy

## Abstract

Drought is a major environmental challenge that significantly limits crop productivity, and its impact varies based on the severity and timing of water scarcity. Soybean [*Glycine max* (L.) Merr.] faces considerable yield constraints under water-deficit conditions. This study evaluated the performance of eight soybean genotypes characterized by different levels of drought tolerance compared with the drought-tolerant world genotype PI416937 under normal [100% of crop evapotranspiration (ETc)] and deficit irrigation (60% ETc) conditions during the 2021 and 2022 seasons at Sakha Agricultural Research Station. Under deficit irrigation, the promising line H4L4 produced 92% (4.07 t/ha) of its productivity under normal irrigation, compared with 89% (2.12 t/ha) for the drought-tolerant genotype PI416937 in an average of two seasons. Applying deficit irrigation saved 37.54% and 38.61% of applied irrigation water across two seasons, whereas genotype H4L4 achieved the highest crop water use efficiency (0.95 and 0.90 kg seeds/m^3^) in the respective seasons, highlighting its potential for sustainable production under water-limited conditions. The promising line H4L4 also exhibited the highest stability and adaptability for seed yield across diverse environments, as confirmed by GGE biplot analysis. Furthermore, the drought susceptibility index (DSI) proved the superiority of H4L4 followed by PI416937, Giza 22, and DR101 for drought tolerance. Additionally, anatomic studies highlighted that PI416937 and H4L4 exhibited superior tolerance by maintaining thicker primary and secondary xylem tissues along with better stem and leaf integrity under irrigation levels. These resilient genotypes, thriving under water-deficit conditions, have significant potential as valuable genetic resources for breeding programs to enhance soybean productivity and sustainability. Additionally, H4L4 may be well-suited for widespread cultivation in water-deficit areas.

## 1 Introduction

Soybean [*Glycine max* L.] Merrill is recognized as one of the most essential summer agricultural legumes. It supplies oil, medicinal ingredients, and high-quality protein for both humans and animals (Pagano and Miransari, 2016). Furthermore, soybean may increase soil fertility by fixing atmospheric nitrogen, benefiting the crop that follows (Ngalamu et al., 2013). Thus, one of the most significant goals for increasing soybean output and area is the development of stable, high-yielding genotypes. In 2023, the global soybean planted area was roughly 128 million hectares with a total production of approximately 385 million metric tons (USDA Foreign Agricultural Service, 2023). Meanwhile, Egypt’s soybean cultivated area expanded to over 24,000 hectares, with a total yield of approximately 82,000 metric tons (FAO, 2023).

Drought is a major environmental concern for agricultural productivity around the world, and climate change has exacerbated the problem. Plant’s response to water stress levels varies depending on developmental stage, severity, duration, and variety of genetics (Farooq et al., 2012; Bodner et al., 2015). Water scarcity is the most critical abiotic stress impacting global soybean production, with approximately 40% of crop losses (Wei et al., 2018; Bigolin and Talamini, 2024). It has a substantial influence on soybean yields, and the sustainability index can assist in identifying drought-resistant cultivars and management approaches that could mitigate yield losses (Poudel et al., 2023).

Soybean cultivation in Egypt faces considerable challenges due to the country’s predominantly arid and semi-arid climate. With annual rainfall generally below 130 mm, agricultural activities depend largely on irrigation, mainly from the Nile River. However, water shortages, particularly during drought periods, pose a serious threat to soybean growth and productivity (Naser et al., 2024). Optimized irrigation practices such as adopting deficit irrigation techniques and including regulating irrigation levels based on crop evapotranspiration (ETc), can impact soybean yield and water efficiency. Research indicates that applying irrigation at 100% ETc leads to improved yields and higher water productivity compared to lower irrigation levels (Elmetwalli et al., 2019). The impact of varying irrigation intervals (every 4 and 6 days) on five soybean varieties (Giza 22, Giza 21, Crawford, Giza 111, and Giza 35), grown in sandy soil under a drip irrigation system, revealed that longer irrigation intervals adversely affected plant height, pod count per plant, 100-seed weight, and seed yield. Among the tested varieties, Crawford exhibited a notably positive response to more frequent irrigation, emphasizing the need for proper irrigation scheduling to minimize drought stress (Safina and Abdel-Wahab, 2018). In summary, drought poses a major challenge to soybean cultivation in Egypt, negatively influencing plant growth and yield.

Drought stress causes significant anatomical changes in soybean plants, impacting different tissues and structures. Insights from the transverse section analyses highlight the following: changes in stem structure and changes in vascular development. Cross-sections of soybean stems under drought conditions exhibit alterations in vascular bundle organization, which can affect the plant’s efficiency in transporting water and nutrients (Makbul et al., 2011). In comparison, drought stress caused a reduction of leaf anatomy features like cuticle, adaxial and abaxial epidermis, palisade parenchyma, and mesophyll. This reduction is attributed to changes in leaf cell size and distribution (Carrera et al., 2021). Also, another reduction was observed in the leaf blade, midvein dimension (length and width), average vessel diameter, phloem, and xylem thickness (Desoky et al., 2025).

In Egypt, it is necessary to expand the local planting soybean area to fulfill the increasing demand for this crop. Interest in cultivating soybeans in newly reclaimed areas outside the Nile Valley has recently grown. In these regions, the primary challenge for horizontal expansion is often the lack of sufficient irrigation water that significantly restricts soybean cultivation. Consequently, intensive research should be conducted on creative strategies to increase both planted areas and crop productivity. Developing crop varieties that are tolerant to water stress could be the most cost-effective solution for farming these lands. Developing crop varieties that are tolerant to water stress could be the most cost-effective solution for farming these lands (Masuda and Goldsmith, 2009; Morsy et al., 2022).

However, implementing effective agronomic strategies and selecting drought-tolerant cultivars can help reduce these impacts and improve soybean productivity under water-limited conditions (Seleiman et al., 2021). Yield stability is governed by plant traits such as resistance or tolerance to environmental stressors. Breeders can improve cultivar stability by identifying relevant elements such as genotype-by-environment interactions (GEI) or stability/instability (El Hosary et al., 2023). Stable function requires resistance to both biotic and abiotic stressors, and identifying the underlying causes of GEI is crucial. Therefore, it is crucial to use genotypes that can effectively exploit and utilize available water more efficiently (Basal, 2017; Wijewardana et al., 2019).

In the present study, we aimed to a) screen eight soybean genotypes for water-deficit tolerance and examine the internal effects of drought stress by transverse sections, b) identify the genotype–environment relationship under normal and 60% field capacity irrigation, and d) recommend adapted genotypes for different water-stressed environments using the GGE biplot model.

## 2 Materials and methods

### 2.1 Experiment layout, soil properties, and meteorological data

A field experiment was conducted at Sakha Agricultural Research Station farm (31.0894°N, 30.9444°E), Agricultural Research Center (ARC), during the 2021 and 2022 summer seasons to evaluate eight soybean genotypes under conventional management practices. Prior to the final tillage, the experimental area was tilled three times, and 150 kg/feddan of calcium superphosphate (15.5% P_2_O_5_) was added. Just prior to the first watering following planting, mineral nitrogen fertilizer was administered at a rate of 15 kg N per feddan as ammonium nitrate (33.5% N). Furthermore, before the second irrigation, 50 kg/feddan of potassium sulfate (K_2_O) was applied. Furthermore, recommended farming procedures were followed without the use of pesticides until harvest. Meteorological data for the 2021 and 2022 seasons were acquired from the Agro-meteorological Station at Sakha from (May to October) (**Table 1**). Soil samples for initial chemical and physical properties were collected from different layers of the experimental locations prior to planting and subjected to the following hydrophysico-chemical analysis. Samples were air-dried, crushed through a 2-mm sieve, and thoroughly mixed. The soil’s chemical and physical characteristics were assessed using the previously outlined methodology (Jackson, 1964; Page, 1982; Klute and Dirksen, 1986). **Table 2** displays the results of soil physical and chemical properties in both seasons, as well as the chemical characteristics of irrigation water (**Table 3**). Moisture parameters, field capacity (FC), and permanent wilting point (PWP) were determined using the pressure membrane method according to Klute and Dirksen (1986).

**Table 1:** Climate conditions (rainfall and temperature) during the two summer seasons (2021 and 2022) of the experiment.

Month	Temperature (c)		Relative humidity (%)		Pan evaporation (mm/day)	Rain (mm/day)
	max	min	max	min		
2021 season						
May	33.35	24.80	75.35	41.25	0.89	00
June	32.14	25.52	80.23	50.21	0.87	00
July	37.17	27.98	85.06	50.48	0.86	00
August	34.52	28.31	85.09	48.70	0.75	00
September	32.41	25.09	83.82	48.12	0.76	00
October	29.51	21.5	75.74	59.74	0.49	00
2022 season						
May	30.01	21.82	76.83	44.35	0.71	00
June	33.04	25.71	82.76	50.23	0.75	00
July	33.07	25.92	64.87	77.32	0.79	00
August	34.75	25.91	77.01	71.03	0.75	00
September	32.94	26.12	83.62	55.22	0.65	00
October	28.75	20.81	90.91	60.77	0.32	00

**Table 2:** Some chemical and physical properties of the experimental soil.

Characteristic	2021	2022
(dS/m/25°C) ^*^	2.36	2.32
Ca^++^ (meq L^−1^)	4.95	4.86
Mg^++^ (meq L^−1^)	2.83	2.78
Na^+^ (meq L^−1^)	16.02	15.75
K^+^ (meq L^−1^)	0.17	0.18
Co_3_^−^ (meq L^−1^)	0	0
HCo_3_^−^ (meq L^−1^)	3.63	3.15
Cl^−^ (meq L^−1^)	11.21	11.01
SO_4_^−^ (meq L^−1^)	9.15	9.41
ESP	9.68	9.6
Soil pH ^**^	8.21	8.09
Organic matter %	1.17	1.18
Available N %	43.31	44.87
Available P %	9.34	8.98
Available K %	323	287
Available micro-elements (mg kg^−1^)		
Fe	43.4	42.5
Zn	8.52	8.61
Mn	30.5	30.7
Cd	0.24	0.26
Pb	1.75	1.73
Texture	Clayey	Clayey
FC %	45.12	45.24
PWP %	24.18	24.42
Bulk density (Mg m^−3^)	1.35	1.34
Total porosity (%)	49.06	49.43
IR (cm h^−1^)	0.95	0.96
PR (N cm^−2^)	230	220
CaCo3 %	2.35	2.37

pH, soil reaction (in 1:2.5 soil:water suspension); EC, electrical conductivity (in soil paste extract, soil salinity); ESP, exchangeable sodium percentage; FC, field capacity; PWP, permanent wilting point; IR (cm h^−1^), basic infiltration rate; PR, soil penetration resistance.

*Measured in soil paste extract.

**Table 3:** Chemical characteristics of irrigation water used in the experiment.

Parameters	pH	EC (dS m^−1^)	SAR	Soluble cations (meq L^−1^)				Soluble anions (meq L^−1^)			
				Na^+^	Ca^++^	Mg^++^	K^+^	Cl^−^	CO_3_^−2^	HCO_3_^−^	SO_4_^−2^
Values	8.39	0.56	3.64	3.8	0.95	1.23	0.11	2.66	0	2.64	0.79

EC, electrical conductivity; SAR, Sodium Adsorption Ratio.

### 2.2 Irrigation water and applied treatments

Two irrigation methods were implemented (normal irrigation and 60% field capacity irrigation). Traditional surface irrigation method was applied, and irrigation water was measured using a cut-throat flume (20 × 90 cm) (Early, 1975). Applied water (AW) was calculated as described by Giriappa (1983) as AW = IW + ER + S, where IW is the irrigation water applied, ER is the effective rain, and S is the amount of soil moisture contribution to consumptive use from the shallow ground water table. The contribution of groundwater table (S) as a percentage of the consumptive use was calculated as GWC% = (ETc − SMD)/ETc × 100, where ETc is the crop evapotranspiration and SMD is the soil moisture depletion (Khalifa, 2018). The water consumptive use (CU) was calculated using the equation of Israelson and Hansen (1962), whereas irrigation application efficiency (Ea) in percent for each treatment was obtained by dividing the total water stored in the root zone by the applied irrigation water (Egbebi et al., 2024), as follows:


Ea = Ws/Wd x 100

where Ea is the water application efficiency (%), Ws is the water stored in the effective root zone, and Wd is the water applied to the field plot.

Crop water use efficiency (CWUE) was calculated using the following equation (Abdelkhalek et al., 2015): CWUE = Yield (kg ha^−1^)/Water consumptive use (m^3^/ha).

Field water use efficiency (FWUE) was calculated in kg m^−3^ for different irrigation systems to clarify how much kg yield is produced from 1 m^3^ applied (Abdelkhalek et al., 2015).

A less surface irrigation treatment of 60% of ETc was implemented in the two seasons based on the percentage of ETc, compared with the normal irrigation (100% of ETc). The water requirements were calculated using the evapotranspiration equations acquired.

### 2.3 Soybean genotypes

The studied soybean genotypes were obtained from the Legume Crops Department, Agricultural Research Center in Giza, Egypt. These genotypes included three soybean cultivars and three local promising lines characterized by different levels of drought tolerance compared with the international drought-tolerant genotype PI416937 and the local drought-tolerant line DR101 (**Table 4**).

**Table 4:** The selected eight genotypes under the present study.

Code	Genotype name	Pedigree	Maturity group	Growth type	Origin
G1	Giza 22	Crawford × Forrest	IV	I	FCRI*
G2	H3L110	DR101 × PI416937	V	D	FCRI*
G3	H6L198	Toano × Nena	IV	I	FCRI*
G4	DR101	Selected from Elgin	V	I	FCRI*
G5	H4L4	DR101 × Lamar	IV	I	FCRI*
G6	Giza 111	Crawford × Celest (Commercial)	IV	I	FCRI*
G7	Toano	Ware × Essex	V	D	AES, USA**
G8	PI416937	Exotic from Japan (drought tolerant)	V	D	Japan

The genotype code, name, pedigree, maturity group, and origin.

### 2.4 Experimental design and trait assessment

To evaluate the previous soybean genotypes under two irrigation treatments, an experiment was designated and sown on May 1 in the 2021 and 2022 seasons. The split-plot design was applied as an experimental design with three replicates in both seasons. The two aforementioned irrigation treatments were distributed randomly in the main plots, whereas the eight soybean genotypes were assigned randomly in the sub-plots. Each plot (harvest area) contained four ridges, each 3 m long and 60 cm apart. Three weeks following planting, plant thinning was carried out to leave two or three plants per hill. The area’s traditional cultural techniques for growing soybeans were followed except for irrigation. In each plot, days to maturity (DM; day, number of days from sowing to 95% of matured pods) and seed yield plot^−1^ (kg) were gathered on the plot base to calculate seed yield (ton ha^−1^), while 10 guarded plants (random samples) were taken to measure plant height (cm, measured from the soil to the tip of the main steam at harvest time), number of branches plant^−1^ (number of primary branches on the main steam at harvest time), number of pods plant^−1^ (total number of pods/plant was recorded at harvest time), and 100-seed weight (g), as well as protein and oil percentage (Williams, 1984; Cunniff and Chemists, 1995; Nouroozi et al., 2015). The seed chemical composition of the studied genotypes was assessed at the Seed Technology Department, Sakha Agricultural Research Station. To evaluate protein content, a certain weight of the finely crushed seeds (approximately 0.1 g) was digested using a micro-Kjeldahl apparatus with 98% H_2_SO_4_ and 30% H_2_O_2_. The crude protein was calculated by multiplying the total nitrogen by 6.25, according to Sanful and Darko (2010). To assess oil content, 10 g of crushed seeds was used to extract the seed oil using petroleum ether for 6 h in the Soxhlet system according to the American Oil Chemists’ Society (AOCS) method (American Oil Chemists Society, 1998). For anatomical assessment, samples of approximately 1 cm of the median internodes of the soybean main stem and its leaf at the age of 30 days were fixed for 48 h in a mixture of FAA (10 mL formalin, 5 mL glacial acetic acid, 35 mL distilled water, and 50 mL ethanol alcohol 95%), followed by washing with 50% ethanol and dehydrating in butanol series. After the dehydration steps, samples were embedded in paraffin wax. Sections (20 μm) were taken using Leica microtome RM2125, stained with erythrothin and crystal violet, and mounted in Canada balsam (Nassar and El-Sahhar, 1998). Photomicrographs of sections were taken and measured using the Leica Light Image Analysis system (LEICA DM750, Wetzlar, Germany) with a digital camera (LEICA ACC50 HD, Germany) at Research Park (CURP) Faculty of Agriculture, Cairo University. The main stem data recorded were stem and pith diameter, and the thickness of the following tissues: epidermis, cortex, primary and secondary phloem, secondary and primary xylem, and primary and secondary vessel diameters. The leaf blade measurements were the thickness of the leaf blade, upper epidermis, mesophyll, palisade & spongy, and lower epidermis tissues and palisade cell diameters.

### 2.5 Statistical analysis

Regular variance analyses of split-plot designs were performed for each experiment in accordance with Gomez and Gomez (1984) guidelines. Then, prior to the combined analysis, Bartlett (1937) method was used to examine the homogeneity of individual error components. The drought susceptibility index (DSI) was evaluated for seed yield t ha^−1^ using the formula DSI = Ys/Yp, where, Ys is the seed yield of the genotype in a stress environment and Yp is the yield of the genotype under non-stress conditions across the two sowing dates (Grzesiak et al., 1996; Peirone et al., 2018). Furthermore, stability analyses using the GGE biplot technique, which combines two concepts (Gabriel, 1971), were used to examine the multi-environment yield trials (MEYT) data (Yan et al., 2007). The method employed a biplot to display the variables (genotype and genotype-by-environment interaction) as a source of variation. This study used genotype-focused scaling to visualize genotypic and environmental comparisons. Furthermore, symmetric scaling offered the best portrayal of the MEYT yield data’s which-won-where pattern (Yan and Rajcan, 2002).

## 3 Results

### 3.1 Effect of irrigation levels on soil characteristics

**Figure 1:**
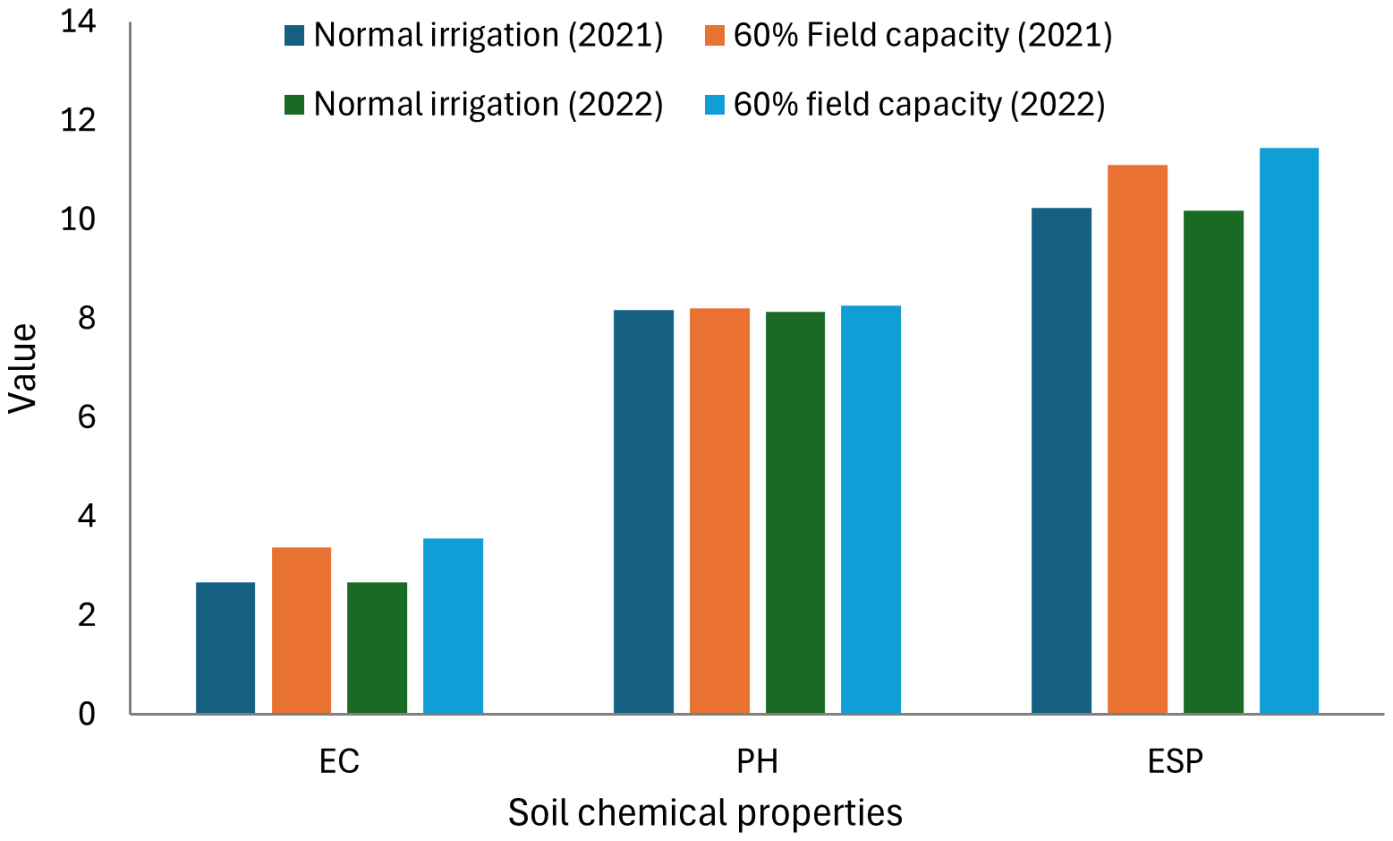
Salinity (EC), soil reaction (pH), and exchangeable sodium percentage (ESP) of the soil as affected by irrigation levels at harvest.

Salinity was measured in saturated soil paste extract as described and presented in **Figure 1**. Ammonium chloride was employed to estimate the exchangeable sodium, subsequently quantified using a flame photometer. Salinity increased under 60% field capacity when compared to normal irrigation, and non-significant differentiation was observed among the two applied years. Soil bulk density, total porosity, soil penetration resistance (PR), and basic infiltration rate (IR) affected by treatment application are displayed in **Table 5**. No significant differences during the two applied years were observed in soil density, total porosity, soil PR, and basic IR.

**Table 5:** Soil bulk density, total porosity, soil penetration resistance (PR), and basic infiltration rate (IR) as affected by irrigation levels at harvest.

	2021				2022			
Irrigation levels	Bulk density (Mg m^−3^)	Total porosity (%)	PR (N cm^−2^)	IR (cm h^−1^)	Bulk density (Mg m^−3^)	Total porosity (%)	PR (N cm^−2^)	IR (cm h^−1^)
Normal	1.34	49.43	230	0.96	1.32	50.19	235	0.98
60% field capacity	1.36	48.68	240	0.85	1.39	47.55	250	0.81

### 3.2 Water relations

Data in **Table 6** show that less irrigation water saved irrigation water by 37.54% and 38.61% in the two seasons. Regarding actual water consumptive use, it is indicated that the seasonal water consumptive use values were affected by fewer irrigation methods. The valuable values (52.87 and 54.04 cm) were obtained from a normal irrigation system, whereas the constringed values (35.34 and 34.90 cm) were obtained under 60% field capacity irrigation methods in the two seasons. The uppermost observed water application efficiency was achieved under 60% field capacity (73.92% and 68.69%) in soybean crops, while the lowest percentages (66.92% and 63.83%) were detected under normal irrigation. The applied water efficiency improved by 7% and 5% due to irrigation with less water irrigation system in the two implemented seasons.

**Table 6:** Some water relations affected by the different irrigation levels in 2021 and 2022 seasons.

Season	Irrigation levels	Applied irrigation water (m^3^/ha)	Water saving %	Water consumptive use (cm)	Irrigation application efficiency %	Water stored (m^3^/ha)
2021	Normal	7,594.58	0.00	52.87	66.92	5,082.35
	60% field capacity	4,743.63	37.54	35.34	73.92	3,506.58
2022	Normal	8,153.95	0.00	54.04	63.83	5,204.84
	60% field capacity	5,005.78	38.61	34.90	68.69	3,438.57

### 3.3 Soybean water use efficiency

CWUE for soybean was significantly affected by irrigation levels and genotypes (**Table 7**). A highly significant effect of irrigation levels was observed on water use efficiency (CWUE and FWUE). The uppermost value of CWUE was achieved in H4L4 (1.05 and 0.99 kg seed/m^3^) under 60% field capacity irrigation in the two seasons, whereas the minimal numbers were recorded under genotype Toano (0.38 and 0.39 kg seed/m^3^) under normal irrigation in the two seasons. However, the obtained results revealed that H4L4 had the best value of FWUE (0.79 and 0.70 (kg seed/m3) under less water irrigation across two seasons of assessment. Meanwhile, less value from FWUE (0.27 and 0.26 kg seed/m^3^) was found in genotype Toano under normal irrigation across the two applicable seasons.

**Table 7:** Crop (CWUE) and field (FWUE) water use efficiencies as affected by the irrigation levels and soybean genotypes and their interaction in 2021 and 2022 seasons.

Irrigation levels	Genotype	CWUE		FWUE	
		2021	2022	2021	2022
Normal	Giza 22 (G1)	0.70	0.63	0.49	0.42
	H3L110 (G2)	0.44	0.50	0.30	0.33
	H6L198 (G3)	0.77	0.78	0.54	0.53
	DR101 (G4)	0.47	0.53	0.33	0.35
	H4L4 (G5)	0.84	0.80	0.59	0.53
	Giza 111 (G6)	0.75	0.70	0.53	0.47
	Toano (G7)	0.38	0.39	0.27	0.26
	PI416937 (G8)	0.41	0.47	0.29	0.32
60% field capacity	Giza 22 (G1)	0.94	0.87	0.70	0.61
	H3L110 (G2)	0.52	0.61	0.39	0.42
	H6L198 (G3)	0.84	0.85	0.63	0.60
	DR101 (G4)	0.61	0.73	0.46	0.52
	H4L4 (G5)	1.05	0.99	0.79	0.70
	Giza 111 (G6)	0.81	0.89	0.61	0.62
	Toano (G7)	0.46	0.42	0.34	0.30
	PI416937 (G8)	0.56	0.63	0.42	0.45
F test		**	**	**	**
LSD 0.05		0.075	0.129	0.053	0.017

CWUE, crop water use efficiency; FWUE, field water use efficiency.

(** indicate p < 0.01).

### 3.4 Effect of irrigation levels on agronomic traits

**Table 8** presents the mean performance of genotypes for all evaluated traits under both normal and 60% field capacity irrigation conditions in the two seasons. The evaluated soybean genotypes manifested a wide variation for all assessed traits across various environments. Days to maturity of soybean genotypes ranged from 112.33 days for the GIZA 22 genotype under 60% field capacity to 143 days for the PI416937 genotype under normal irrigation. According to the recorded data, genotypes GIZA 22 and GIZA 111 disclosed the earliest genotypes. Regarding plant height, the topmost values for plant height were observed in genotype H3L110 followed by genotype H6L198 across all applied environments. The water shortage declined the plant height in the H3L110 genotype from 128.67 to 123.60 cm and from 126 to 121.33 cm in seasons 2021 and 2022, respectively. This deficiency in plant height recorded 3.8% on average due to decreasing water irrigation in the two seasons. Furthermore, the PI416937 genotype measured the shortest plants in the first season (2021, under both normal and 60% field capacity conditions), whereas in the second season (2022), the Toano genotype presented the least plants in height under both irrigation regimes (**Table 8**). This reduction reached 7.15% in the PI416937 genotype and 18.38% in the Toano genotype under drought stress.

**Table 8:** Mean performance of soybean studied traits as affected by the interaction of irrigation levels and genotypes in 2021 and 2022 seasons.

Irrigation levels	Genotype	Maturity date (day)		Plant height (cm)		No. of branches plant^−1^		No. of pods/plant^−1^	
		1st	2nd	1st	2nd	1st	2nd	1st	2nd
Normal	Giza 22 (G1)	118.33	121.67	109.00	105.00	4.27	3.87	127.73	123.60
	H3L110 (G2)	137.33	134.33	128.67	126.00	4.07	4.10	77.47	79.07
	H6L198 (G3)	125.00	124.33	122.00	120.00	4.20	4.53	117.67	121.07
	DR101 (G4)	141.00	133.67	87.00	79.00	5.07	5.73	92.73	89.53
	H4L4 (G5)	126.67	122.00	97.00	87.67	2.60	2.80	130.87	134.20
	Giza 111 (G6)	125.33	121.00	108.00	118.00	4.60	4.27	117.33	125.60
	Toano (G7)	140.00	137.00	80.00	74.33	5.13	3.70	82.47	89.33
	PI416937 (G8)	143.00	140.00	70.00	76.00	3.53	2.67	89.47	78.07
60% field capacity	Giza 22 (G1)	112.33	114.00	101.00	93.33	3.47	3.60	111.07	117.20
	H3L110 (G2)	135.67	131.33	123.60	121.33	3.60	2.93	61.53	73.47
	H6L198 (G3)	122.00	121.67	113.00	109.07	2.20	3.73	109.60	108.93
	DR101 (G4)	138.67	131.33	77.93	66.00	3.80	4.03	85.27	81.52
	H4L4 (G5)	124.67	120.33	83.67	85.00	1.87	2.07	112.47	115.47
	Giza 111 (G6)	118.67	112.67	100.93	103.00	3.87	3.80	102.60	117.47
	Toano (G7)	131.67	129.33	74.00	60.67	4.47	3.20	78.20	75.93
	PI416937 (G8)	139.33	137.33	65.00	65.33	2.93	2.20	75.93	69.47
F test		**	**	**	**	**	**	**	**
LSD 0.05		2.37	3.17	2.63	3.12	0.73	1.32	6.57	6.83
Irrigation levels	Genotype	100-Seed weight (g)		Seed yield (ton ha^−1^)		Protein (%)		Oil (%)	
Normal	Giza 22 (G1)	18.99	17.96	3.74	3.40	29.72	30.28	22.42	23.36
	H3L110 (G2)	17.56	15.30	2.32	2.71	26.53	26.75	24.33	25.34
	H6L198 (G3)	16.55	17.59	4.11	4.27	30.30	30.80	21.78	22.61
	DR101 (G4)	21.88	18.89	2.48	2.89	30.81	31.10	22.22	24.01
	H4L4 (G5)	18.22	16.96	4.48	4.36	28.80	28.80	22.17	23.32
	Giza 111 (G6)	18.91	16.74	3.99	3.80	30.05	31.00	25.39	26.69
	Toano (G7)	18.36	16.85	2.02	2.14	30.68	31.50	20.29	21.81
	PI416937 (G8)	17.43	16.47	2.18	2.58	25.26	24.51	18.25	19.31
60% field capacity	Giza 22 (G1)	17.75	15.83	3.34	3.07	33.02	33.46	19.01	19.87
	H3L110 (G2)	17.17	14.17	1.86	2.13	32.75	33.70	19.64	19.95
	H6L198 (G3)	15.90	15.45	2.99	2.99	35.25	36.35	17.22	18.73
	DR101 (G4)	20.59	17.20	2.19	2.59	34.56	35.51	16.57	17.40
	H4L4 (G5)	17.45	14.87	4.15	3.99	35.68	36.00	17.83	17.16
	Giza 111 (G6)	17.80	15.24	2.89	3.13	32.30	32.80	16.28	16.75
	Toano (G7)	17.97	15.15	1.64	1.47	33.90	33.85	17.86	18.69
	PI416937 (G8)	15.48	15.42	2.01	2.23	34.88	35.20	19.30	18.75
F test		**	*	**	**	**	**	**	**
LSD 0.05		1.56	0.65	1.33	0.17	1.48	1.95	1.57	2.92

* and ** indicate p < 0.05 and p < 0.01, respectively.

In the same manner, the number of branches per plant in the eight soybean genotypes displayed significant variation among genotypes under normal irrigation and 60% field capacity. The best number of branches plant^−1^ was detected in the DR101 and Toano lines under normal and shortage irrigation in the second and first seasons, respectively. Exploring the number of pods plant^−1^ (**Table 8**) revealed a decrease in the assessed trait with a decrease in the water supplementary. The uppermost number of pods plant^−1^ was found in the H4L4 genotype (130.87–134.20) under regular irrigation and (112.47–115.47) across less water irrigation.

When going forward and evaluating the yield-related traits under normal irrigation and 60% field capacity in the two seasons, 100-seed weight and seed yield ton/ha displayed a variable differentiation among assessed genotypes in the two seasons of evaluation in addition to the regular and water-deficit situations. The DR101 genotype gave the best 100-seed weight in irrigation treatment in the two seasons. Continued evaluation of the yield-related traits and measuring seed yield ton/ha showed significant differentiation between the estimated genotypes under the various applied conditions. Genotype H4L4 was the most stable and the highest producer of seed yield t ha^−1^ across all the environments.

The related seed quality traits were estimated for the assessed soybean genotypes. Protein and oil content as percentages were employed. Protein content (**Table 8**) in the eight evaluated genotypes varied from 36.35% in the H6L198 line under 60% field capacity in the second season to 24.51% in the PI416937 line (under normal conditions in the second season). Similarly, oil content was differentiated among genotypes under the applied water regime in the two years of assessment. The maximum oil percent was assigned to GIZA 111 (26.69%) under normal irrigation in the second year, whereas the minimized value was observed in the same genotype (16.28%) under 60% field capacity in the first year.

### 3.5 Genotype adaptability and stability

#### 3.5.1 The “which-won-where” view of the GGE biplot

**Figure 2:**
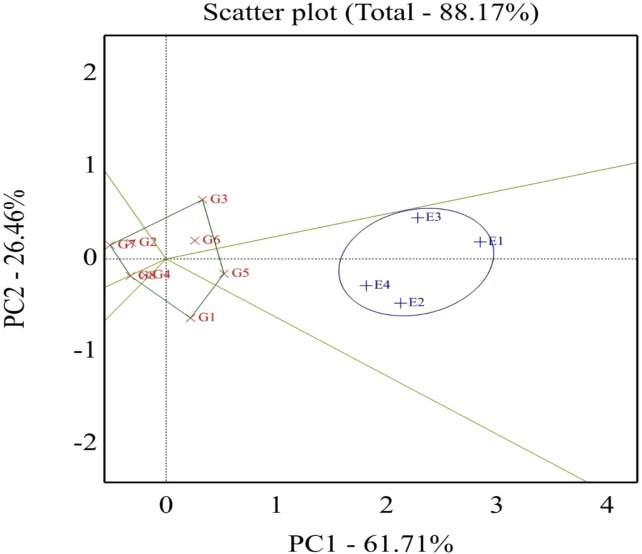
Polygon view of the GGE biplot for which-won-where pattern of eight soybean genotypes grown across four environments. G1, GIZA 22; G2, H3L110; G3, H6L198; G4, DR101; G5, H4L4; G6, GIZA 111; G7, Toano; G8, PI416937. Environments: E1, normal irrigation in the first season; E2, 60% field capacity irrigation in the first season; E3, normal irrigation in the second season; E4, 60% field capacity in the second season.

The best genotypes for each environment were evaluated using GGE biplot analysis mega-environments (which-won-where, **Figure 2**). Genotypes located on the vertex of a polygon are the best or poorest genotypes in some or all environments, except the bottom left quadrant. This enables the researcher to have a specific and valid justification to recommend genotypes that are good for a particular environment. This also means that any genotypes can be tested in those few mega-environments and still good yield results can be obtained. The GGE biplot also can give information for researchers to make a decision and conclusion about specific correlations among environments and genotypes. The GGE biplot model accounted for 88.17% of the total variation distributed as 61.71% and 26.46% of variance attributable to the first (PC1) and second (PC2) principal components, respectively. Which-won-where or which-is-best for what analysis represented that the top-performing genotypes were positioned at the peaks of the polygon, so the ideal genotype H4L4 (G5), followed by H6L198 (G3), was the winner in the whole environment. These results manifested that these genotypes had a high yield, and their behaviors were superior in all environments. Genotype G5 disclosed the top average seed yield (large PC1 scores) and ranked the first genotypes in all environments, whereas genotypes G2, G7, and G8 registered the lowest mean values (PC1 scores <0) for seed yield.

**Figure 3:**
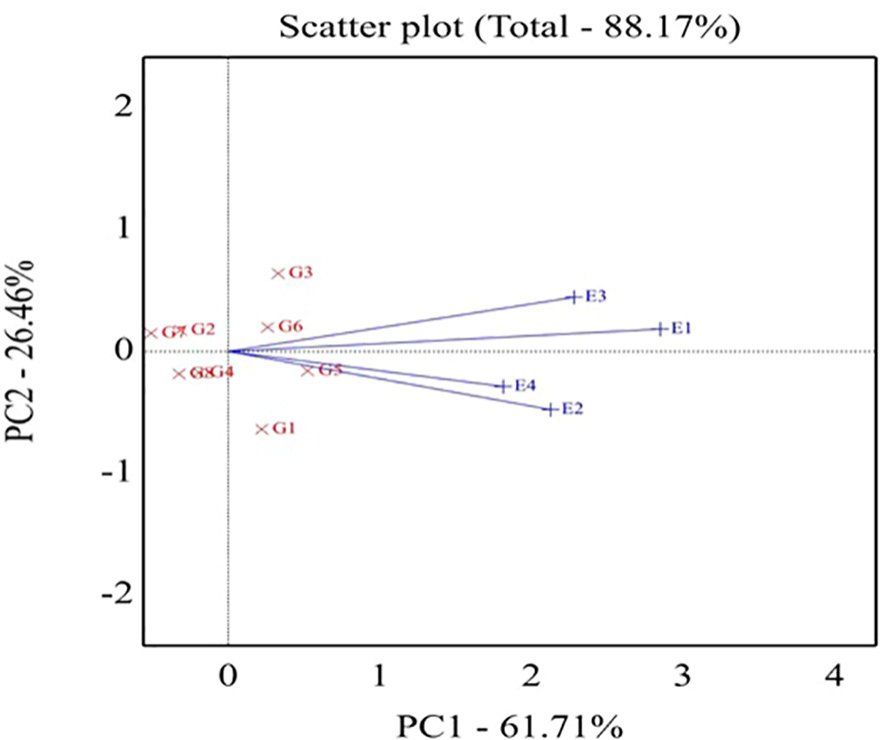
Biplot of relationships among the four tested environments. G1,GIZA 22; G2, H3L110; G3, H6L198; G4, DR101; G5, H4L4; G6, GIZA111; G7, Toano; G8, PI416937. Environments: E1; normal irrigation inthe first season; E2; 60% field capacity in the first season; E3; normalirrigation in the second season; E4; 60% field capacity in thesecond season.

In the same context, **Figure 3** explores genotypes and environments in the GGE biplot in the same plot. The angle between environment vectors provides information on the correlation among environments. An acute angle indicates a positive correlation, a right angle specifies no correlation, and a negative correlation is related to an obtuse angle. Thus, a positive correlation was declared between the E1 and E3 environments in addition to the E2 and E4 environments.

**Figure 4:**
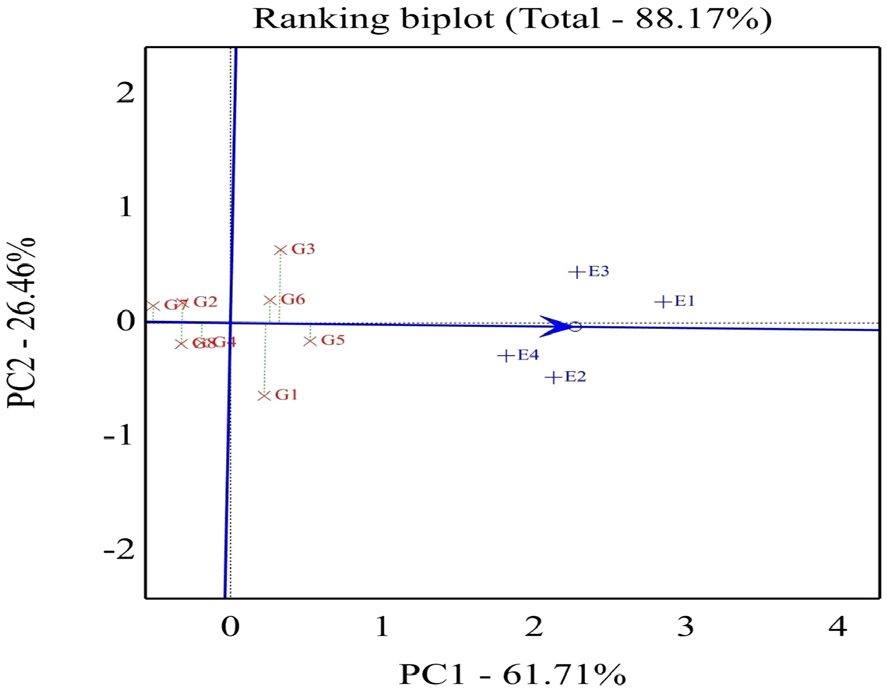
Biplot of relationships among the four tested environments and thestability of assessed eight soybean genotypes. G1, GIZA 22; G2,H3L110; G3, H6L198; G4, DR101; G5, H4L4; G6, GIZA 111; G7,Toano; G8, PI416937. Environments: E1, normal irrigation in the firstseason; E2, 60% field capacity irrigation in the first season; E3,normal irrigation in the second season; E4, 60% field capacityirrigation in the first season.

As shown in **Figure 4**, the percentage of total variation of the two-way interaction reached 88.17%, indicating the goodness of fit and validity of the GGE biplot method. The straight line with a single arrow (abscissa) that passes through the biplot origin is referred to as the average environment coordinate (AEC). The arrow direction points to higher mean performance for genotypes. The small circle spotted on this line represents the average of environment PC1 and PC2 scores. It is defined by the average coordinates of all tested environments in the biplot. However, the line (ordinate) passes through the biplot origin and is perpendicular to the AEC line, indicating the stability proper. Thus, the genotype located closer to the AEC line in the two directions had a more stable yield and vice versa. Consequently, the genotypes with a mean above the average are shown in a descending pattern of G5 < G3 < G6 < G1. Meanwhile, the other genotypes exhibited a mean less than the average and showed a descending pattern of G4 < G2 < G8 < G7.

**Figure 5:**
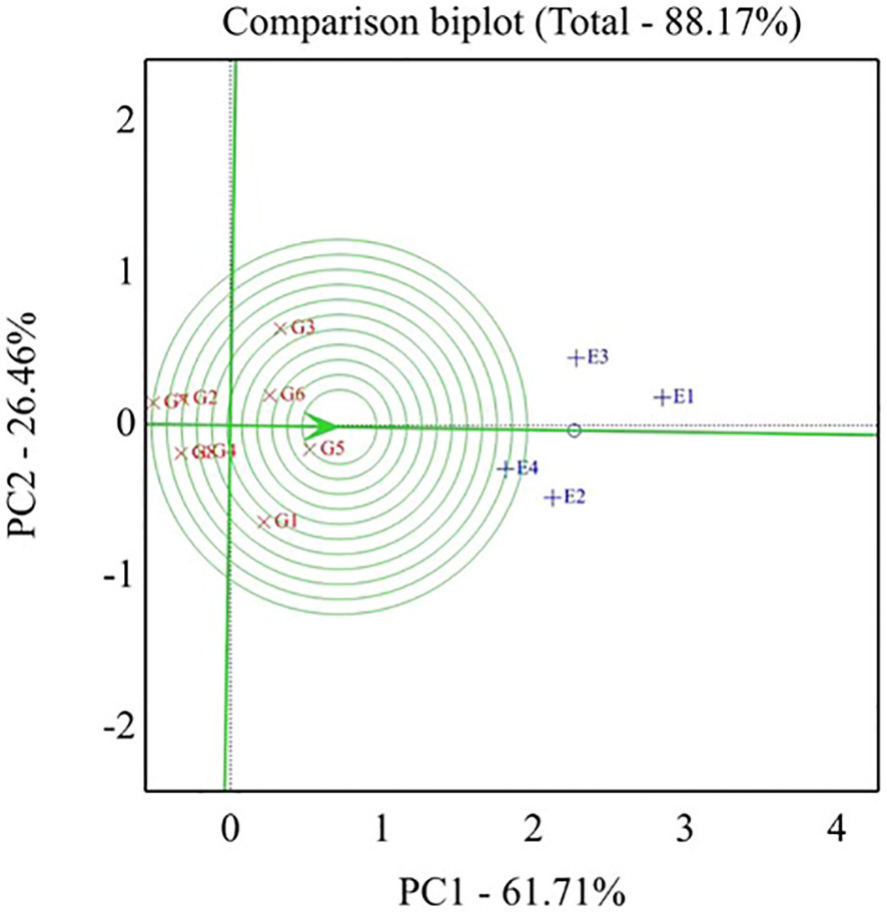
GGE biplot, ideal genotypes for seed yield (t ha-1) of eight soybean genotypes across four tested environments. G1- GIZA 22, G2- H3L110, G3- H6L198 , G4- DR 101, G5- H4L4, G6- GIZA 111, G7- TOANO, G8- PI416937. Environments: E1- normal irrigation in the first season, E2- 60% field capacity irrigation in the first season, E3- normal irrigation in the second season, and E4- 60% field capacity irrigation in the first season.

Concerning the stable genotype, regardless of the seed weight, genotype G5 was located very close to the AEC line reflecting its topmost average stability, while genotypes G1 and G3 showed minimum average stability based on its slightly placed away from AEC abscissa. In conclusion, the length of the average environment vector was sufficient to select genotypes based on yield mean performance. The ideal genotype should exhibit both high mean performance and stability, meaning it performs well across all environments. This is characterized by a long vector length among high-yielding genotypes and minimal GEI. The ideal genotype is H4L4-G5 (positioned closest to the AEC arrow), indicating its superior yield potential and stability compared to other genotypes (**Figure 5**). Additionally, genotypes located near the outermost concentric circles can range from desirable to undesirable, such as Toano (G7). In plant breeding programs, the goal is to identify and select the most promising genotypes for specific target environments.

#### 3.5.2 Drought susceptibility index

**Figure 6:**
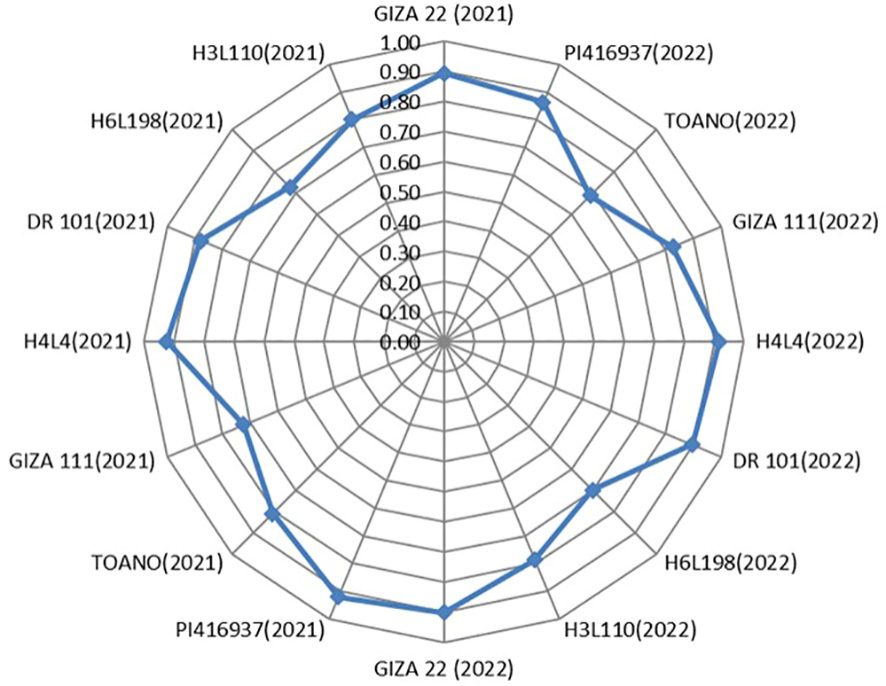
Drought susceptibility index of the eight soybean genotypes for seed yield (t ha-1) in the two seasons of assessment (2021 and 2022).

For screening soybean genotypes according to their tolerance and susceptibility to drought, the DSI was applied as an appropriate parameter to achieve this task. Seed weight (t ha^−1^) in both evaluated seasons for the eight genotypes expressed significant differences between normal and 60% field capacity environments at each season. The supreme DSI for seed weight was given by H4L4, PI416937, Giza 22, and DR101 in the two seasons of evaluation (2021 and 2022). Meanwhile, the highly sensitive genotype was H6L198 in both seasons, while the other genotypes had moderate resistance to drought (**Figure 6**). Remarkably, genotypes H4L4, PI416937, Giza 22, and DR101 can be planted under the condition of water shortage and/or used in the breeding program to donate genes related to drought tolerance.

### 3.6 Anatomical studies

#### 3.6.1 Stem anatomical

**Figure 7:**
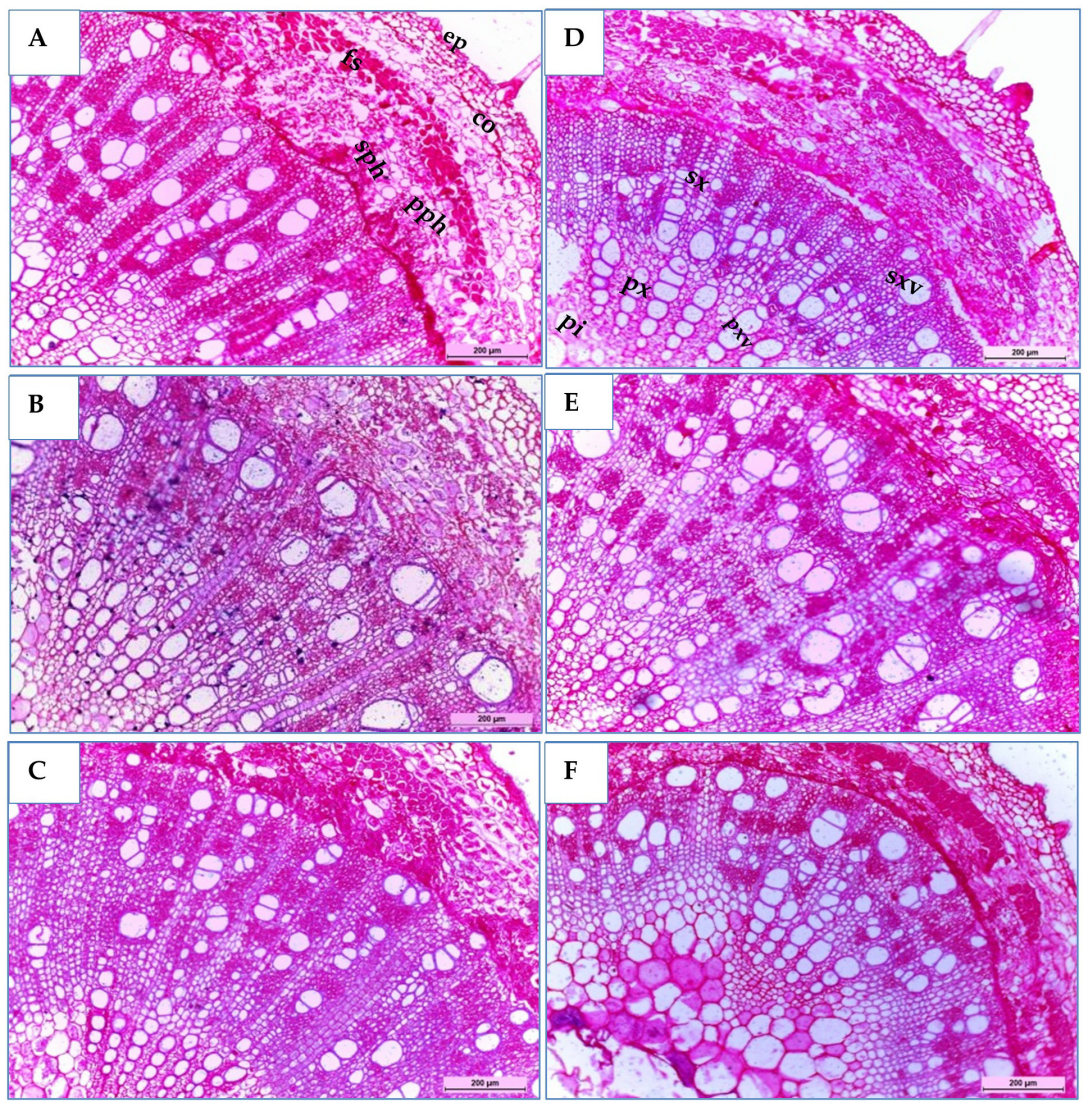
Transverse sections of a magnified portion of the medial internode in the main stems of soybean genotypes, H4L4 **(A, D)**, PI4I6937 **(B, E)**, and Toano **(C, F)** under two water treatments; normal irrigation **(A–C)** and drought stress **(D–F)**. Tri,Trichomes; ep, epidermis; co, cortex; fs, fiber strands; pph, primary phloem; sph, secondary phloem; sx, secondary xylem; sxv, secondary xylem vessels; px, primary xylem; pxv, primary xylem vessels; pi, pith.

Three soybean genotypes (H4L4, PI416937, and Toano) were selected to study their stem anatomical structures under normal and drought stress conditions. As shown in **Table 9** and **Figure 7**, the transverse microscopic sections displayed differences in measurements and counts of certain stem anatomical features among unstressed and stressed circumstances. In the three soybean genotypes, the outline of the middle internode of the soybean main stem appeared surrounded by a monolayer of epidermal cells, followed by approximately five layers of oval cortex cells with intercellular spaces. Sclerenchyma tissue spread as continuous layers above stressed plant phloem, while in unstressed plants, it seemed like grouped layers. Sclerenchyma tissue in plants suffering from hydric stress provides a protective adaptive advantage. As a result of secondary growth, sieve elements of the primary phloem appeared tangentially comprised. The vascular cylinder formed a continuous ring consisting of an outer secondary phloem and an inner secondary xylem, followed by the primary xylem, whereas the pith occupied the central part of the stem. Notably, H4L4 revealed the highest primary and secondary phloem and xylem tissue thickness and primary xylem vessel diameter under normal irrigation (**Table 9**). Regarding the effect of drought stress in the three genotypes, it caused a decrease in most stem anatomical measurements, which varies from one genotype to another. In general, stem diameter and its internal tissues declined under drought stress. Compared to those in unstressed plants, the reduction percentages in stem diameters were 1.33%, 2.78%, and 48.53% for H4L4, PI416937, and Toano, respectively. Additionally, the pith diameter increased slightly by 10.96% in H4L4 and decreased by 9.47% and 37.49% in PI416937 and Toano, respectively. In addition, the epidermal layer decreased by 18.39%, 25.19%, and 44.73%, while cortex thickness minimized by 5.43%, 16.28%, and 27.29% in H4L4, PI416937, and Toano, respectively. Mostly, the pith parenchyma cells of the normal plants appeared smaller than those of stressed ones. The pith parenchyma cells appeared bigger in H4L4 and PI416937, while they were damaged in Toano. Notably, trichomes appeared clearly on the H4L4 epidermal layer, which reduced water loss (**Figures 7A, D**). Moreover, the H4L4 genotype obtained the lowest deficiency percentages of all studied stem anatomical traits under drought stress followed by PI416937, but the highest deficiency percentages were obtained by Toano (the sensitive genotype).

**Table 9:** Measurements of the main stem and leaf in microns of certain histological features of the middle portion in three soybean genotypes (H4L4, PI416937, and Toano) under normal irrigation (N) and 60% field capacity (S).

Mean of the main stem measurements (µ)									
Characters	H4L4			PI416937			Toano		
	N	S	change %	N	S	Change %	N	S	change %
Main stem diameter	5,523.9	5,450.6	−1.33	5,490.9	5,338.2	−2.78	6,468.5	3,329.1	−48.53
Pith diameter	2,667.2	2,959.5	10.96	2,331.1	2,110.2	−9.48	2,576.4	1,610.6	−37.49
Epidermis thickness	17.83	14.55	−18.40	21.87	16.36	−25.19	22.02	12.17	−44.73
Cortex thickness	153.98	145.62	−5.43	206.7	173.04	−16.28	145.19	105.57	−27.29
Fiber thickness	105.24	117.45	11.60	95.92	86.91	−9.39	75.03	64.57	−13.94
Primary phloem thickness	55.66	49.41	−11.23	55.48	53.24	−4.04	41.28	33.21	−19.55
Secondary phloem thickness	90.34	74.6	−17.42	85.05	64.67	−23.96	39.62	35.56	−10.25
Secondary xylem thickness	672.18	403.31	−40.00	563.73	834.05	47.95	652.29	334.01	−48.79
Secondary vessel diameter	62.24	71.86	15.46	94.49	92.07	−2.56	49.63	48	−3.28
Primary xylem thickness	252.42	243.52	−3.53	222.65	213.02	−4.33	163.23	166.09	1.75
Primary vessel diameter	66.67	65.74	−1.39	62.11	37.28	−39.98	39.74	44.57	12.15
Mean of the leaf midrib measurements (µ)									
Leaf midvein thickness	704	768.87	9.21	618.57	448.24	−27.54	504.4	467.7	−7.28
Leaf main vascular bundle dimension									
Length	292.47	249.15	−14.81	185.7	129.72	−30.13	164.77	149.15	−9.48
Width	230.04	200	−13.06	206.2	104.7	−49.23	184.62	100.73	−45.44
Xylem tissue thickness	155.34	142.13	−8.50	114.1	106.25	−6.90	78.29	61.52	−21.42
Xylem vessel diameter	31.78	26.95	−15.20	19.87	18.83	−5.23	18.66	14.14	−24.22
Phloem tissue thickness	44.96	51.46	14.46	49.08	29.47	−39.96	42.11	22.22	−47.23
Mean of the leaf blade measurements (µ)									
Leaf blade thickness	189.51	147.82	−22.00	168	151.9	−9.57	104.9	129.08	23.05
Upper epidermis thickness	14.18	12.94	−8.74	19.37	15.59	−19.51	14.14	11.05	−21.85
Palisade tissue thickness	65.12	52.32	−19.66	66.65	62.77	−5.82	48.68	40.82	−16.15
Spongy tissue thickness	88.97	60.51	−31.99	54.55	45.45	−16.68	56.84	48	−15.55
Lower epidermis thickness	16.05	16.6	3.43	11	12.88	17.09	11.8	10.88	−7.80

± % changes = percentage of increase or decrease to normal irrigation.

#### 3.6.2 Leaf anatomical

**Figure 8:**
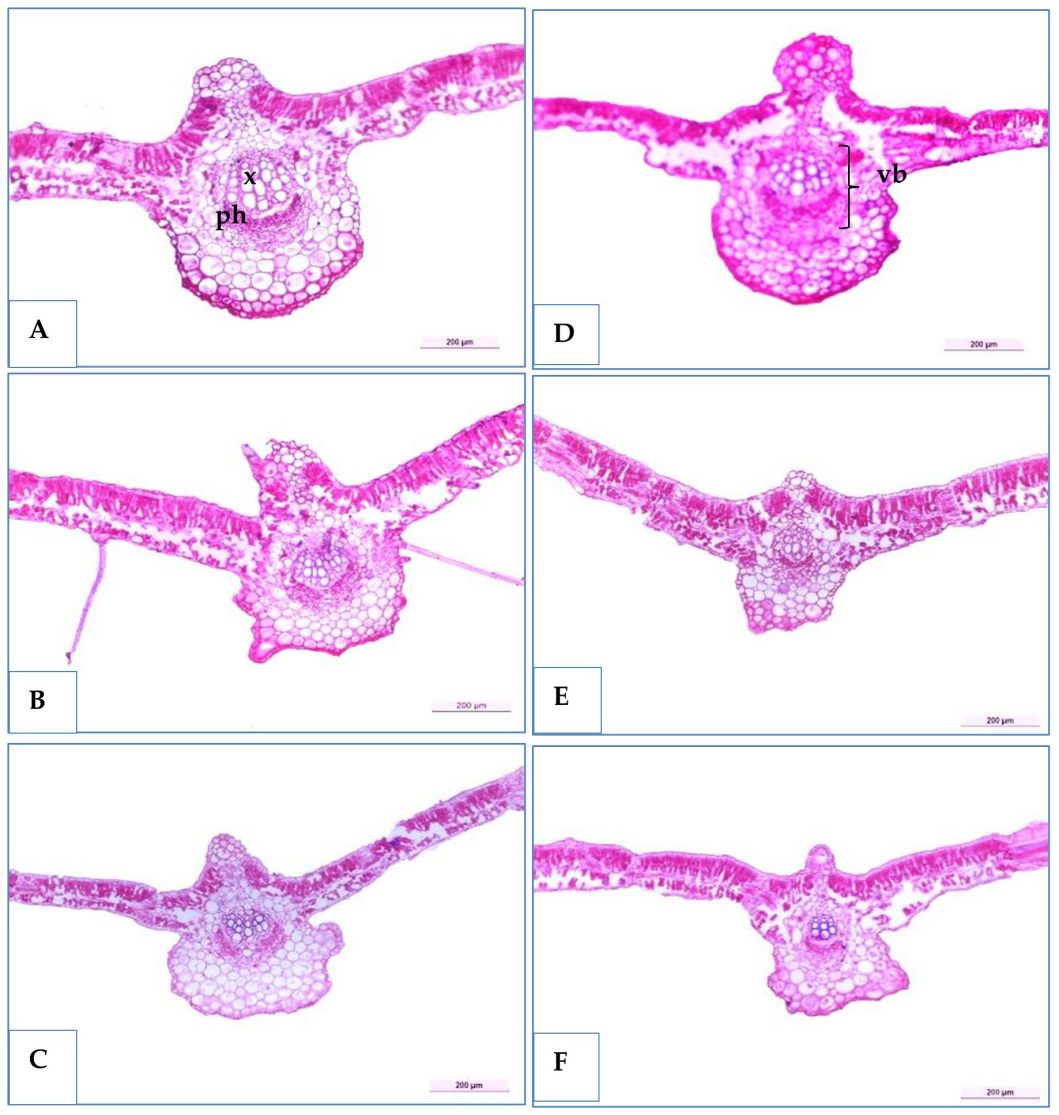
Transverse sections of some soybean genotypes leaves (H4L4, PI4I6937, and Toano) under two water treatments; normal irrigation **(A–C)** and drought stress **(D–F)**. x, xylem; ph, phloem; mvb, main vascular bundle.

**Figure 9:**
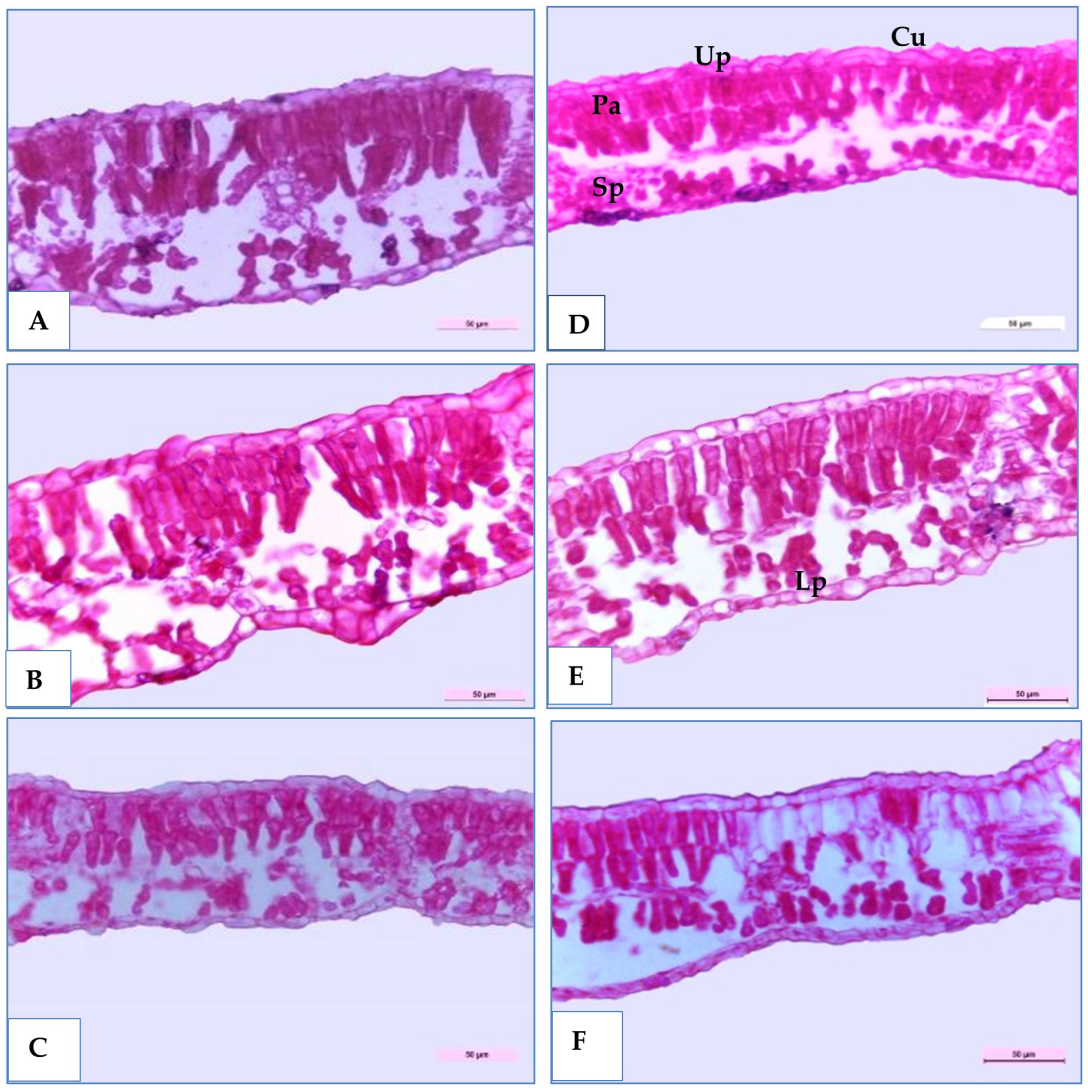
Transverse sections of magnified portion in the medial portion of some soybean genotypes leaves blade (H4L4, PI4I6937 and Toano) under two water treatments; normal irrigation **(A–C)** and drought stress **(D–F)**. Cu, Cuticle; Up, Upper epidermis; Pa, Palisade tissue; Sp, Spongy tissue; Lp, Lower epidermis.

Three soybean genotypes (H4L4, PI416937, and Toano) were selected to study their anatomical structures in cross-sections under normal and water deficiency, as shown in **Table 9** and **Figures 8** and **9**. The obtained results showed that all sectioned genotypes under normal irrigation had well-organized rectangular epidermal cells with stomata, which clearly appeared in the upper and lower epidermal cells. Furthermore, they had typically columnar palisade and spongy parenchyma cells with intercellular spaces. The results in **Table 9** and **Figure 8** disclosed that 60% field capacity had a harmful effect on soybean leaf anatomical measurements in the three genotypes. The reduction of leaf anatomical feature values was in midvein thickness, main vascular bundle dimension (length and width), xylem and phloem tissue thickness, and xylem vessel diameter. Compared to that of the unstressed plants, the leaf midvein thickness of PI416937 and Toano reduced by 27.54% and 7.27%, respectively, whereas in H4L4, it increased by 9.21%. Moreover, both xylem tissue thickness and xylem vessel diameters were diminished by 8.50%, 6.89%, and 21.42% in xylem thickness and by 15.19%, 5.23%, and 24.22% in xylem vessel diameters and for H4L4, PI416937, and Toano, respectively. Also, another reduction was observed in the leaf blade and its internal tissues. Compared to that of unstressed plants, the leaf blade thickness of H4L4 and PI416937 reduced by 21.99% and 9.56%, respectively, and that of Toano increased by 23.05%. The palisade tissue condensed by 19.65%, 5.82%, and 16.15%, respectively, and the spongy tissue lowered by 31.98%, 16.68%, and 15.55% for H4L4, PI416937, and Toano, respectively (**Figure 9**). Furthermore, under drought stress, the H4L4 and PI416937 genotypes showed the thickest epidermal tissue with the biggest cuticle layer. In addition, the palisade cells were denser, more compactly arranged, and wider in diameter, when compared to the Toano genotype under drought stress. Notably, under drought stress, H4L4 gave the highest leaf tissue measurements compared with other genotypes.

## 4 Discussion

Soybean [*G. max* (L.) Merr.] is the most-grown high-value legume crop with immense economic significance (Shaheen et al., 2016; Yan et al., 2020; Arya et al., 2021). Drought was predicted to be responsible for approximately 50% of the soybean yield decline. Developing drought tolerance in cultivars is a critical step toward lowering yield losses and sustaining crop output under drought circumstances (Specht et al., 1999; Wei et al., 2018).

Water shortage significantly influences the plant height of genotypes, impacting their growth and development in various ways. It caused a decline in leaf area, plant height, pod production, 100-seed weight, seed yield, harvest index, and other parameters (Ohashi et al., 2006; Tang et al., 2017). This occurs due to inhibited cell elongation and reduced internode elongation under water-deficit conditions. The severity of height reduction can vary depending on the duration and intensity of drought stress. Soybean water stress is the most severe during the blooming stage and the time post-flowering. As a result, it is required to assess the response of soybeans with different genetic germplasms to drought stress at various phases of growth (Fahad et al., 2017; Arya et al., 2021; Poudel et al., 2023).

Different soybean genotypes exhibit varying degrees of height reduction under drought stress. Some genotypes may maintain relatively normal height with minimal reduction, while others may experience more pronounced stunting (Zhi-Hui et al., 2003; Quansah et al., 2020). Drought induces physiological responses in soybean plants that contribute to reducing plant height. These responses include altering hormone levels (such as abscisic acid and cytokinins), changes in nutrient uptake and distribution in addition to alterations in gene expression related to flowering and maturation pathways, and adjustments in metabolic processes to cope with water scarcity (Muhammad Aslam et al., 2022; Shaffique et al., 2023; Li et al., 2024). Likewise, drought stress influences yield components such as seed quantity per plant, seed size, and pod number. Genotypes differ in their ability to preserve these components under drought conditions. Some may show reduced seed number but better seed size, while others may have more significant yield reductions (Gebre et al., 2022).

The effectiveness of drought tolerance traits can vary under different environmental conditions. Some genotypes perform well under controlled drought conditions but may not be effective under natural field conditions, highlighting the importance of genotype × environment interactions (Mwiinga et al., 2020; Rani et al., 2023). In this regard, it has been observed that water stress during flower formation results in a shorter flowering period, leading to fewer flowers and pods, and ultimately a reduced number of seeds per plant (Basal, 2017). Previous studies by Mimi et al. (2017) and Fernández García et al. (2020) have also reported a decline in all agronomic traits due to water stress.

In the present study and under deficit irrigation, line H4L4 was considered the promising genotype and maintained 92% of its productivity. It achieved the highest crop water use efficiency, stability, and adaptability for seed yield across diverse environments, indicating its potential for sustainable production under water-limited conditions. Poudel et al. (2023) characterized the water deficit on 10 cultivars. They reported that drought decreased the seed number by 45% and the seed weight by 35%. Hossain et al. (2024) displayed that the G00001 genotype had the uppermost 100-seed weight (14.60 g) and seeds pod^−1^ (1.90) under water-shortage conditions when compared with other genotypes, whereas the BD2333 genotype manifested the maximum decrease in pod number plant^−1^ (85.90%), plant height (37.10%), relative water content (34.40%), and carotenoids (56.70%) under drought compared to normal conditions. Similarly, Chen et al. (2020) evaluated 136 soybean genotypes for drought tolerance. They identified 26 varieties as drought tolerant such as S14, S93, and S135 with high yield (Y > 300 kg) and drought-tolerant index (DTI) >1.3. Moreover, the oil content decreased by 2.9%, whereas protein content was minimized by 4.4% under water scarcity during seed fill (Dornbos and Mullen, 1992). Furthermore, the mean seed protein content under water shortage decreased by 4.5%, while seed oil content measured by slight increment reached 1.2% (Šarčević et al., 2022).

GGE biplot analysis is currently a comprehensive technique that can graphically address the majority of questions concerning genotype-by-environment tables (Yan, 2001; Yan and Rajcan, 2002; Yan et al., 2007). Farshadfar and Sadeghi (2014) described “GGE” as the combination of the genotype main effect (G) and genotype interaction as two sources of variation in the site regression (SREG) model. Assessing the sustainability index for drought in soybeans is critical for various reasons, particularly when considering the increased frequency and severity of drought conditions caused by climate change.

Studying the which-won-where pattern of multi-environment yield trials is important for the possible existence of different mega–mega-environments in a region (Yan, 2001). The polygon view of a biplot is the best way to visualize the interaction patterns between genotypes and environments and to effectively interpret a biplot (Yan et al., 2007). Concerning **Figure 6**, the rays divided the biplot into four sectors, and the environments filled into one of them. A good feature in the view of the GGE biplot is that the top genotype for each sector has a higher yield than the others in all environments (Yan and Rajcan, 2002). The current results are in a parallel line with those obtained by Dehghani et al. (2010, 2013). Different winning genotypes in different environments were also reported by Bhartiya et al. (2017) and Vaezi et al. (2017).

Drought stress caused a reduction in most anatomical features of plant stem (Shafqat et al., 2021). Transverse sections are a powerful strategy for studying the internal effects of drought stress on plants. They give precise information about cell and tissue structural changes, vascular development, and overall plant adaptations. By examining these cross-sections, researchers may gain a better understanding of how drought impacts various plant genotypes and develop drought-tolerance strategies for crops. Our anatomic studies highlighted that PI416937 and H4L4 had superior tolerance by maintaining thicker primary and secondary xylem tissues along with better stem and leaf integrity under irrigation levels. The reduction in the stressed plants may be due to the reduction that occurred in all tissue cell sizes and intercellular spaces, which is because of the lower water content in the water-deficient plants (Gonçalves et al., 2017). Moreover, sclerenchyma tissue in plants suffering from hydric stress afforded a protective adaptive advantage, so it increased in thickness in response to drought stress (Canne-Hilliker and Kampny, 1991). In addition, xylem development is suppressed by drought, so the xylem tissue thickness and xylem vessel diameter are reduced under drought stress compared with unstressed plants. Microscopic observation of stem cross-sections showed that H4L4 and PI416937 have some characteristics such as a thick cuticle and epidermal layer, which are desirable because they reduce the rate of transpiration. Also, the presence of trichomes and big parenchyma pith cells are the main storage tissue that increases water storage capacity. Higher secondary xylem thickness and vessel diameters were associated with increased capacity and water utilization of conductive tissues (Lupo and Moshelion, 2024). In comparison, the sensitive genotype (Toano) had the thinnest epidermal layer, secondary xylem, destroyed pith cells, and a smaller number of vessels and appeared to have plugged xylem vessels. These factors may explain the sensitivity of this genotype to drought. Vollenweider et al. (2016) reported that the xylem is often plugged by amorphous materials that fill the cell wall.

Regarding the effect of drought on the leaf blade, it reduced leaf lamina thickness, which occurred due to reduced cell volume and intercellular spaces, allowing cells to continue being more juxtaposed (De Souza et al., 2021). Under limited water availability, leaf blades of H4L4 and PI416937 had thicker upper and lower cuticles that minimized non-stomata water loss. In this respect, Yang et al. (2021) declared that the cuticle is a type of lipid membrane that improves plant drought resistance by obstructing and reducing water evaporation. Furthermore, these two genotypes exhibited wider and denser palisade cells under water stress compared with Toano. Mesophyll tissue with few intercellular spaces represents an adaptive benefit in high photosynthetic capacity plants (Acosta-Motos et al., 2017).

## 5 Conclusions

The promising line H4L4 recorded the best values in the number of pods/plant and seed yield (ton/ha), whereas days to maturity was assigned to the Giza 22 genotype (less value is desirable). H4L4 appeared more adaptable and stable when GGE biplot analyses were implemented. Otherwise, the DR101 genotype disclosed the maximum number of branches/plant and 100-seed weight, whereas the H6L198 and H3L110 genotypes revealed the maximum protein and oil percentages across all environments. Finally, part of these genotypes may play a vital role in the breeding program and soybean production.

## Data Availability

The raw data supporting the conclusions of this article will be made available by the authors, without undue reservation.
